# Dietary practices, food purchasing, and perceptions about healthy food availability and affordability: a cross-sectional study of low-income Malaysian adults

**DOI:** 10.1186/s12889-022-12598-y

**Published:** 2022-01-28

**Authors:** Chee Wen Eng, Shiang Cheng Lim, Carrie Ngongo, Zhi Hao Sham, Ishu Kataria, Arunah Chandran, Feisul Idzwan Mustapha

**Affiliations:** 1 RTI International, Selangor, Malaysia; 2 Center for Global Noncommunicable Diseases, RTI International, Seattle, USA; 3 Center for Global Noncommunicable Diseases, RTI International, New Delhi, India; 4 Disease Control Division, Ministry of Health, Putrajaya, Malaysia

**Keywords:** Dietary practice, Community health, Behavioral health, Urban poor, Low-income adults, Sugar-sweetened beverages, Industrialised foods

## Abstract

**Background:**

Malaysia has the highest rate of overweight and obesity among Asian countries. Obesity is increasing particularly among low-income populations. This study aimed to assess dietary practices among low-income adults in urban communities, including gender and ethnic variation, to inform the development of locally tailored, evidence-based interventions for health promotion.

**Methods:**

This cross-sectional study was conducted from August to December 2020. Stratified sampling was employed to recruit 2983 low-income adults from households in the bottom 40% of the economic spectrum (B40) at six public, low-cost housing flats in the Federal Territory of Kuala Lumpur, Malaysia. Face-to-face interviews were conducted using a structured questionnaire to understand dietary practices, perceptions of healthy food availability and affordability, and factors affecting food purchasing decisions.

**Results:**

A staggering 89.5% of B40 adults were found to not consume adequate daily amounts of fruits and vegetables. In addition, 68.1% reported consuming sugar-sweetened beverages at least once per week, including commercially packed ready-to-drink beverages, sugar-added self-prepared drinks, and premixed drinks. Intake was statistically significantly higher among men (71.7%), Malays (70.3%), and Indians (69.9%). Bread and other commercially baked goods were the most common processed foods, and 52.9% of respondents consumed it at least once per week. Majorities reported that healthy foods were moderately available and priced. The top three reported factors affecting food purchase choices were price (79.4%), availability (75%), and taste (73%).

**Conclusions:**

Adults in low-cost housing communities have unhealthy dietary patterns with low intake of fruits and vegetables and high intake of ultra-processed foods and calorie-dense local foods, with variations across gender and ethnicity. The study highlighted the need for educating low-income families on diet-disease relationships and possibilities for inexpensive, healthy eating that rely on minimally processed fresh foods. Policymakers engaging the food industry are advised to consider how to increase the affordability and availability of healthy foods in low-income communities in urban areas.

**Supplementary Information:**

The online version contains supplementary material available at 10.1186/s12889-022-12598-y.

## Background

Obesity imposes a significant burden of morbidity and mortality on worldwide populations, driving up the risk of noncommunicable diseases (NCDs) including type 2 diabetes and cardiovascular diseases. Overweight and obesity are now on the rise in low- and middle-income countries, particularly in urban settings [1, 2]. Malaysia, a multi-ethnic upper middle-income country, has the highest rate of overweight and obesity in Asia [3, 4]. In 2019, 30.4% of Malaysian adults were overweight and 19.7% were obese based on the World Health Organization (WHO) classification of Body Mass Index [5]. One consequence is the increasing prevalence of diabetes among adults, which rose from 11.2 to 18.3% between 2011 and 2019 [5, 6]. Hypertension prevalence remains high at 30% [5].

Rapid urbanization, modernization, and adoption of a lifestyle with reduced physical activity and increasing intake of calories have resulted in rising obesity rates [7]. Age, gender, locality, and social characteristics produce variations in the prevalence of obesity [8, 9]. A study in the Federal Territory of Kuala Lumpur, the national capital and largest city in Malaysia, found that the proportion of obesity among the urban poor was significantly higher than the national average (29.9% vs. 17.7%) and that monthly household income was negatively associated with obesity [10]. In addition, low-income people have lower health literacy than the national population as a whole [5].

The Better Health Programme Malaysia is a part of the United Kingdom Foreign, Commonwealth and Development Office’s Prosperity Programming to address the growing burden of NCDs. The Programme was co-created with the Malaysian Ministry of Health (MOH) to focus on the urban poor communities that are disproportionately affected by the negative environmental factors that increase NCDs risks [11]. In this programme, the poor are classified as B40, having the lowest 40% of household incomes [12]. In 2019, Kuala Lumpur residents were members of the B40 group if their monthly household income was RM9,150 (~USD2,200) or less [13]. The Better Health Programme Malaysia used the concentration of high-density, public, low-cost housing as a proxy to target urban poor populations. We sought to assess dietary practices and variation across gender and ethnicity to inform the development of locally tailored, evidence-based interventions for behavioural change to prevent obesity and NCDs.

## Methods

### Study design and sites

This cross-sectional study was carried out among the residents of the low-cost high-rise flats of six People’s Housing Programme [*Program Perumahan Rakyat* (PPR) *or Perumahan Awam* (PA) in local language] locations in Kuala Lumpur. The People’s Housing Programme is a government social public housing initiative to resettle squatters and provide housing for low-income households. The study locations were (i) PPR Pekan Kepong Setia, (ii) PA Sri Negeri Sembilan, (iii) PPR Wahyu, and (iv) PPR Fasa 8/9 Bandar Baru Sentul in Kepong district, and (v) PA Seri Melaka, and (vi) PA Seri Kota in Cheras district.

### Participants and sampling method

We adopted a door-to-door recruitment approach involving in-person contact to identify eligible respondents. We included respondents who i) were 18 years old or above, ii) residents of the selected low-cost high-rise flats, and iii) were willing and able to provide written informed consent. The sample size was calculated using a formula for a population-based descriptive study based on the prevalence of various outcome indicators (taken as 50% to get maximum sample size), a margin error of 5%, a confidence interval of 95%, and a response rate of 80%, leading to an estimated sample size of 2880 from six study locations (480 respondents per PPR). Within the estimated sample size, we disaggregated further to facilitate equitable representation by gender and age groups (supplementary Table 1). The age-wise proportional allocation of sample relied on the Kuala Lumpur state data from Population and Housing Census of Malaysia 2010 report [14].

### Data collection

All interviewers underwent thorough training on the study protocol, data collection methods, study questionnaire, data confidentiality, as well as safety procedures with respect to COVID-19 guidelines. In total, we approached 3437 residents. Among them, 2983 were eligible and participated in the study, resulting in a response rate of 86.8%. We collected data through face-to-face interview by using a structured questionnaire. The development of the questionnaire was informed by a literature review of the existing evidence and Malaysia’s National Health and Morbidity Survey [5, 15]. The questionnaire was developed in English, then translated to Malay and Mandarin. Prior to formal data collection, we tested the multilingual questionnaire in a pilot study involving 33 adults in low-income communities to ensure that the content and language used were accurate, easily understood and culturally appropriate. Through the interviews, we collected the respondents’ sociodemographic data and ascertained their consumption of fruits and vegetables as a marker of healthy diet whilst unhealthy diets included ultra-processed foods, beverages included sugar-sweetened beverages (SSBs), and common local foods whose preparations usually involve large amounts of oil, sugar, and/or salt. We defined adequate consumption of fruits and vegetables as five servings per day in accordance with the WHO recommendations. Ultra-processed foods were defined within the context of the NOVA food classification system, which groups foods or beverages according to the nature, extent, and purpose of industrial processing [16, 17]. Ultra-processed foods included (i) commercially baked goods (including cookies, pizza dough, breads like burger buns and pastries), (ii) packaged snack foods (including crackers, popcorn, chips, candies, chocolate and biscuits) and (iii) fast food such as KFC and McDonalds. SSBs included commercially packed ready-to-drink beverages (including carbonated and non-carbonated drinks such as fruit juices, sport and energy drinks, and flavoured beverages), premixed drinks (including instant drink products containing sugar such as premixed coffee, tea, chocolate, soy, and cereal), and sugar-added self-prepared drinks (including coffee, tea, chocolate, or malted beverages with added sugar and/or sweetened condensed milk/creamer). We also included less healthy local foods such as (i) Street fried foods (banana fritters, fish crackers, and curry puffs), (ii) dessert foods including traditional sweets such as *kuih bakar* (pandan custard cake)*, bingka ubi* (baked tapioca cake)*, and kuih talam* (pandan-coconut layered cake). Respondents reported consumption frequency in the categories of “≥ once a day”, “≥ once a week” and “seldom/never.” We further asked about meal preparation practices and eating out, as well as perceptions of availability and affordability of healthy food products.

### Data analyses

We performed descriptive analyses to describe respondents’ sociodemographic characteristics, dietary practices, perceptions on availability and price of healthy food, and food purchasing factors. The categorical variables were illustrated in number and percentage distribution, while the continuous variable (age) was described by median and interquartile range (IQR) due to right-skewed distribution. We used Pearson’s Χ^2^ test to compare the differences in characteristics and behaviours between participants of different gender and ethnic groups. The statistical significance level was set at 0.05. All analyses were performed using Stata version 14 (StataCorps LP, Texas, USA).

## Results

### Sociodemographic characteristics of respondents

A total of 2983 respondents participated in this study. As shown in Table 1, male and female respondents were evenly balanced across the Malay, Indian, and Chinese ethnic groups. The majority of respondents were Malay (64.4%), married (60.8%) and had attained higher secondary education (64%). Most respondents worked in the private sector (46.3%) while 9.6% were unemployed, and 5.2% were students. About half of the respondents (49.8%) reported an average monthly household income of under RM3,000 (~USD720), with no significant differences by gender or ethnicity. The majority (68.1%) had an average household size of 3 to 5 people. Household size was significantly higher among Malay respondents as compared with other ethnic groups. Compared to male respondents, female respondents were more likely to be married (64.3%), work as homemaker or unpaid worker (30.5%) and have a higher education level (pre-university and above, 20.2%). Compared to Malay and Indian respondents, Chinese respondents were older (median age of 45 years) and more likely to be married (67.2%), retired (10.9%) or unemployed (11.3%).

**Table 1:** Sociodemographic characteristics of low-income adults by gender and ethnic groups

Variables	Total (*N* = 2983)	Gender		Ethnicity		
		Male (***n*** = 1486)	Female (***n*** = 1497)	Malay (***n*** = 1920)	Chinese (***n*** = 485)	Indian (***n*** = 565)
	**n (%)**					
Age, median years (IQR)	36 (26–49)	36 (25–49)	36 (27–49)	34 (25–46)	45 (33–56)*	36 (24–48)
Age group, years						
≤ 30	1149 (38.5)	578 (38.9)	571 (38.1)	797 (41.5)	112 (23.1)	235 (41.6)
31–50	1211 (40.6)	587 (39.5)	624 (41.7)	778 (40.5)	208 (42.9)	219 (38.8)
> 50	623 (20.9)	321 (21.6)	302 (20.2)	345 (18.0)	165 (34.0)*	111 (19.7)
Gender						
Male	1486 (49.8)	–	–	958 (49.9)	253 (52.2)	271 (48.0)
Female	1497 (50.2)	–	–	962 (50.1)	232 (47.8)	294 (52.0)
Ethnicity						
Malay	1920 (64.4)	958 (64.5)	962 (64.3)	–	–	–
Chinese	485 (16.3)	253 (17.0)	232 (15.5)	–	–	–
Indian	565 (18.9)	271 (18.2)	294 (19.6)	–	–	–
Other	13 (0.4)	4 (0.3)	9 (0.6)	–	–	–
Marital status						
Unmarried	1035 (34.7)	586 (39.5)	449 (30.0)	675 (35.2)	124 (25.6)	230 (40.7)
Married	1812 (60.8)	849 (57.1)*	963 (64.3)*	1177 (61.3)	325 (67.2)*	303 (53.6)
Divorced/widowed	135 (4.5)	50 (3.4)	85 (5.7)	68 (3.5)	35 (7.2)	32 (5.7)
Education						
Primary school or below	174 (5.8)	73 (4.9)	101 (6.8)	71 (3.7)	70 (14.4)*	32 (5.7)
Lower secondary school^a^	367 (12.3)	174 (11.7)*	193 (12.9)*	201 (10.5)	79 (16.3)*	87 (15.4)
Higher secondary school^b^	1909 (64.0)	1009 (67.9)*	900 (60.1)*	1289 (67.1)	256 (52.8)	357 (63.2)
Pre-university or above	533 (17.9)	230 (15.5)*	303 (20.2)*	359 (18.7)	80 (16.5)	89 (15.8)
Occupation						
Government employee	114 (3.8)	73 (4.9)	41 (2.7)	98 (5.1)*	4 (0.8)	12 (2.1)
Private sector employee	1381 (46.3)	836 (56.3)	545 (36.5)	886 (46.2)	207 (42.7)	278 (49.2)
Self-employed	404 (13.6)	241 (16.2)	163 (10.9)	274 (14.3)	65 (13.4)	65 (11.5)
Homemaker/unpaid worker	479 (16.1)	23 (1.6)*	456 (30.5)*	295 (15.4)	80 (16.5)	102 (18.1)
Retired/pensioner	161 (5.4)	118 (8.0)	43 (2.9)	91 (4.8)	53 (10.9)*	17 (3.0)
Unemployed	285 (9.6)	117 (7.9)	168 (11.2)	183 (9.6)	55 (11.3)*	47 (8.3)
Student	156 (5.2)	77 (5.2)	79 (5.3)	90 (4.7)	21 (4.3)	44 (7.8)*
Average monthly household income (RM)						
< 3000	1485 (49.8)	731 (49.2)	754 (50.4)	930 (48.4)	246 (50.7)	302 (53.5)
3000–4999	1183 (39.7)	608 (40.9)	575 (38.4)	794 (41.4)	188 (38.8)	198 (35.0)
≥ 5000	315 (10.6)	147 (9.9)	168 (11.2)	196 (10.2)	51 (10.5)	65 (11.5)
Household number						
1–2 person	347 (11.6)	178 (12.0)	169 (11.3)	198 (10.3)	85 (17.5)*	60 (10.6)
3–5 person	2031 (68.1)	1021 (68.7)	1010 (67.5)	1281 (66.7)	356 (73.4)	386 (68.3)
6–8 person	561 (18.8)	270 (18.2)	291 (19.4)	408 (21.3)*	44 (9.1)	109 (19.3)
> 8 person	44 (1.5)	17 (1.1)	27 (1.8)	33 (1.7)	0	10 (1.8)

Chi-square test or Wilcoxon rank-sum test (age) was conducted, a *p*-value of lesser than 0.05 (*) indicates significant difference between gender or among ethnic groups

*IQR* Interquartile range, *RM* Ringgit Malaysia

^a^ Completion of Lower Secondary Assessment Examination or equivalent in secondary school in Malaysia, indicates about 9 years of formal education

^b^ Completion of Malaysian Higher School Certificate or equivalent in secondary school in Malaysia, indicates about 11 years of formal education

### Dietary practices

Most respondents (89.5%) had inadequate intake of fruits and vegetables (Table 2). Inadequate intake was highest among the Malay (90.5%), followed by Indian (89.7%), and Chinese (85.2%). A significantly higher proportion of females indicated that they cooked or prepared meals at home (81.8%), while male respondents were more likely to report that they dined out at least once per week (53.1%). Intake of various types of SSBs was common, with more than two-thirds of the respondents (68.1%) reporting consumption of SSBs at least once per week. Weekly SSBs consumption was significantly higher among males (71.7%), Malays (70.3%), and Indian (69.9%). The most frequently consumed SSBs were self-prepared drinks with sugar followed by premixed drinks and soft drinks. Compared to other ethnic groups, Malays were more likely to consume self-prepared drinks with sugar (20.2%) and premixed drinks (6.1%) more than once a day. The most commonly consumed ultra-processed food was commercially baked goods, especially among women, Malay, and Indian respondents. Malay respondents were also more likely than others to report frequently consuming street fried food or local desserts.

**Table 2:** Dietary practices among low-income adults by gender and ethnicity

Variables	Total (*N* = 2983)	Gender		Ethnicity		
		Male (***n*** = 1486)	Female (***n*** = 1497)	Malay (***n*** = 1920)	Chinese (***n*** = 485)	Indian (***n*** = 565)
	**n (%)**					
Prevalence of inadequate intake of fruit or vegetables^a^	2670 (89.5)	1335 (89.8)	1335 (89.2)	1738 (90.5)*	413 (85.2)	507 (89.7)
Cook/prepare meals at home	1539 (51.6)	315 (21.2)	1224 (81.8)*	1028 (53.5)	229 (47.2)	272 (48.1)
Dining out at least once in a week^b^	1382 (46.3)	789 (53.1)	593 (39.6)*	920 (47.9)	207 (42.7)	250 (44.3)
**Frequency of ultra-processed beverages and foods consumption**						
Overall intake of SSBs at least once per week^c^	2031 (68.1)	1066 (71.7)*	965 (64.5)	1349 (70.3)*	280 (57.7)	396 (69.9)*
CPRD drinks						
≥ Once a day	57 (1.9)	37 (2.5)*	20 (1.3)	39 (2.0)	10 (2.1)	8 (1.4)
≥ Once a week	571 (19.2)	344 (23.3)*	227 (15.2)	350 (18.3)	88 (18.2)	131 (23.3)
Seldom/never	2343 (78.9)	1098 (74.2)	1245 (83.5)	1523 (79.7)	385 (79.7)	424 (75.3)
Sugar-added self-prepared drink						
≥ Once a day	540 (18.1)	275 (18.5)	265 (17.7)	388 (20.2)*	40 (8.3)	107 (18.9)*
≥ Once a week	1233 (41.4)	657 (44.3)*	576 (38.5)	821 (42.8)	191 (39.5)	219 (38.8)
Seldom/never	1206 (40.5)	552 (37.2)	654 (43.8)	708 (36.9)	253 (52.3)	239 (42.3)
Premixed drinks						
≥ Once a day	165 (5.6)	72 (4.9)	93 (6.2)	116 (6.1)	20 (4.2)*	28 (5.0)
≥ Once a week	633 (21.2)	332 (22.4)	301 (20.2)	387 (20.2)	126 (26.3)*	119 (21.2)
Seldom/never	2175 (73.2)	1076 (72.7)	1099 (73.6)	1415 (73.8)	334 (69.6)	415 (73.8)
Commercially baked goods						
≥ Once a day	238 (8.0)	97 (6.6)	141 (9.5)*	156 (8.2)	33 (6.8)	48 (8.5)*
≥ Once a week	1333 (44.9)	660 (44.7)	673 (45.1)	887 (46.5)*	185 (38.2)	253 (45.0)
Seldom/never	1396 (47.1)	719 (48.7)	677 (45.4)	865 (45.3)	266 (55.0)	261 (46.5)
Packaged snack foods						
≥ Once a day	77 (2.6)	40 (2.7)	37 (2.5)	54 (2.8)	8 (1.7)	15 (2.7)
≥ Once a week	861 (29.0)	460 (31.0)	401 (27.0)	564 (29.5)	132 (27.3)	162 (28.9)
Seldom/never	2029 (68.4)	982 (66.3)	1047 (70.5)	1291 (67.6)	344 (71.1)	385 (68.5)
Fast food						
≥ Once a day	28 (0.9)	17 (1.2)	11 (0.7)	19 (1.0)	3 (0.6)	5 (1.1)
≥ Once a week	445 (15.0)	229 (15.5)	216 (14.5)	270 (14.1)	88 (18.4)	86 (15.2)
Seldom/never	2496 (84.1)	1234 (83.4)	1262 (84.8)	1631 (84.9)	388 (81.0)	473 (83.7)
Frequency of less healthy local food consumption						
Fried foods						
≥ Once a day	106 (3.6)	60 (4.1)	46 (3.1)	90 (4.7)*	6 (1.2)	10 (1.8)
≥ Once a week	1142 (38.5)	571 (38.5)	571 (38.4)	799 (41.8)*	157 (32.6)	180 (32.1)
Seldom/never	1722 (58.0)	852 (57.5)	870 (58.5)	1025 (53.6)	319 (66.2)	371 (66.1)
Dessert food						
≥ Once a day	97 (3.3)	47 (3.2)	50 (3.4)	78 (4.1)*	8 (1.7)	10 (1.8)
≥ Once a week	945 (31.9)	456 (30.9)	489 (32.9)	646 (33.8)*	141 (29.3)	154 (27.5)
Seldom/never	1923 (64.9)	974 (65.9)	949 (63.8)	1186 (62.1)	333 (69.1)	396 (70.7)

Chi-square test was conducted, a p-value of lesser than 0.05 (*) indicates significant difference between gender or among ethnic groups

*CPRD* commercially packed ready-to-drink, *SSBs* sugar-sweetened beverages

^a^ Inadequate fruits or vegetables intake: consume less than a total of 5 servings of fruits and/or vegetables per day according to WHO dietary recommendation for adults

^b^ Dining out for having main meals or snacks i.e. teatime/supper

^c^ Sugar-sweetened beverages include commercially packed ready-to-drink beverages, sugar-added self-prepared drinks, and premixed drinks

### Perceptions on availability and price of healthy food and purchasing factors

Nearly half of the respondents reported that healthy foods were moderately available (Table 3). Compared to Chinese and Indian respondents, Malay respondents were more likely to indicate that healthy food products were easily available. Nearly two-thirds of respondents (59.5%) perceived that healthy food are moderately priced. The most commonly reported factors affecting food purchase choices were price (79.4%), availability (75%), and taste (73%). There was no significant difference between gender among these factors. Across ethnicity, Indian respondents were most concerned about price and least concerned about a food’s nutritional value.

**Table 3:** Perceptions on availability and price of healthy food and factors affecting food purchasing choices among low-income adults by gender and ethnicity

Variables	Total (*N* = 2983)	Gender		Ethnicity		
		Male (***n*** = 1486)	Female (***n*** = 1497)	Malay (***n*** = 1920)	Chinese (***n*** = 485)	Indian (***n*** = 565)
	**n (%)**					
Availability of healthy food						
Easy	1153 (38.8)	575 (38.7)	582 (38.9)	785 (40.9)*	164 (33.8)	204 (36.1)
Moderate	1440 (48.5)	719 (48.4)	727 (48.6)	898 (46.8)	255 (52.6)	287 (50.8)
Available with some difficulties	318 (10.7)	168 (11.3)	152 (10.2)	190 (9.9)	62 (12.8)*	66 (11.7)
Rare/unavailable	59 (2.0)	24 (1.6)	36 (2.4)	47 (2.5)	4 (0.8)	8 (1.4)
Price of healthy food						
Low	712 (23.9)	363 (24.4)	349 (23.3)	432 (22.5)	140 (28.9)*	139 (24.6)
Moderate	1775 (59.5)	901 (60.6)*	874 (58.4)	1152 (60.0)*	275 (56.7)	338 (59.8)
High	496 (16.6)	222 (14.9)	274 (18.3)*	336 (17.5)	70 (14.4)	88 (15.6)
Food purchasing factors^a^						
Price	2358 (79.4)	1164 (78.3)	1207 (80.6)	1535 (80.0)	359 (74.0)*	464 (82.1)
Nutritional value	2017 (67.9)	1000 (67.3)	1025 (68.5)	1320 (68.8)	338 (69.7)*	359 (63.5)
Taste	2168 (73.0)	1094 (73.6)	1083 (72.3)	1417 (73.8)	333 (68.7)	418 (74.0)
Availability	2228 (75.0)	1109 (74.6)	1131 (75.6)	1428 (74.4)	369 (76.1)	431 (76.3)
Popularity	992 (33.4)	517 (34.8)	480 (32.1)	657 (34.2)	144 (29.7)	191 (33.8)

Chi-square test was conducted, a p-value of lesser than 0.05 (*) indicates significant difference between gender or among ethnic groups

^a^Multiple response variables

## Discussion

Healthy eating is recognized as an essential modifiable factor for the prevention and management of obesity. Regular consumption of sufficient amounts of vegetables and fruits are associated with reduced risk of NCDs [18, 19]. An estimated 1.8% of the total global disease burden may be attributed to inadequate levels of fruit and vegetable consumption [20]. In line with other studies locally and in the Southeast Asia region [21–23], the low consumption of fruits and vegetables found in this study alarmingly foreshadow future NCD risk, especially given the established high NCD risk of low-income people in urban environments [24] Fruit and vegetable consumption is associated with income, as lower-income adults are less likely to consume fruits and vegetables than their higher-income counterparts [25–27]. One local study pointed out that a majority of Malaysians were knowledgeable about the source and role of dietary fibre in human health, including its role as a laxative assisting in the reduction of body weight and cholesterol level. However, most of them did not know about the recommended intake amount [28]. The benefits of fruit and vegetable consumption extend beyond fibre content; their vitamin and mineral content also contribute to their documented role in preventing NCDs and lowering the odds of weight gain and obesity [29–32]. Consumption of adequate of fruits and vegetables should be strongly encouraged and advocated.

Ultra-processed foods are food and drink products that are formulated to be industrially manufactured with no or minimal whole foods and produced with food additives and substances that are not commonly used in culinary preparations such as flavours, colours, and sweeteners, on top of salt, sugar, oils and fats [17, 33]. Many studies have consistently showed the association of ultra-processed food intake with obesity and related cardio-metabolic outcomes whilst urbanization, the rise of consumerism, convenience, and aggressive manufacturer marketing strategies all contribute to rising consumption of ultra-processed foods [34–38].

In this study, we found that SSBs were the most consumed within the ultra-processed foods category. The high overall intake of SSBs across gender and ethnicity, echoing findings of the National Health and Morbidity Survey in 2019 that men tend to consume more SSBs and that self-prepared drinks with added sugar are the most commonly consumed SSBs [5]. Beverage and food preferences often vary by ethnicity; culture may be a contributing factor [39–41]. In this study, Malay adults were more likely to consume self-prepared drinks with added sugar, while premixed drinks were most common among Chinese adults, and commercially packed ready-to-drink beverages were most common among Indian adults. Malay adults tended to consume more of all types of ultra-processed food, except for the fast food favored by Chinese adults.

Messaging on avoiding ultra-processed foods and local foods high in sugar, oil, and fat is appropriate for all communities. That said, nutritional guidance interventions should consider the patterns of typical food preparation for different ethnic groups, emphasizing the changes that would be most relevant. Sensitization about unhealthy local foods might particularly target the Malay community, while communications targeting the Chinese community might focus on premixed drinks and fast food. Information on self-prepared drinks with added sugar will be best targeted to the Malay and Indian communities. Women are usually in charge of food preparation at home, underlining their important role in establishing and implementing healthy nutrition in the family [42, 43]. However, studies have also shown that more men are taking up the household dietary gatekeeper role, especially in food purchasing, hence their responsibilities in promoting healthy eating and in family food work should not be neglected or silenced [44, 45]. Food consumption within families is ultimately the product of interactions and negotiations between family members [46, 47]. For greatest impact, efforts to improve food literacy and enhance skills and behaviours necessary to select and prepare healthy foods should target both men and women [47–49]. Family meals should be an educational tool for the acquisition of healthy eating habits, which will have an impact on nutritional behavior of all family members.

While most of the respondents in our study indicated that healthy foods were moderately available and affordable, price was their most important reported consideration for making food choices. Price concerns may contribute to the low consumption of fruits and vegetables and high intake of energy-dense foods including commercially baked goods and SSBs. This aligns with other reports that food selection is not only a behavioural choice, but also an economic one [50]. Low socioeconomic groups generally have a more restricted food budget and may prioritize food and non-food necessities, such as rice, meat, clothing, transportation, and housing over fruits and vegetables. A study in the United States has shown that pricing interventions may have some measurable effects on weight outcomes, particularly among populations with low socio-economic status [51]. Public health strategies and approaches to dietary change for health promotion would do well to take diet costs into account. Community-based policies and interventions may alleviate the burdens faced by budget-constrained families, supporting them to improve the quality of their diets through price changes and income assistance. Education-based interventions may increase the perceived value of healthy eating among the population [52]. One possible option is to introduce a healthy-food inducement program for the population; a program that encourages people to purchase healthy foods items by providing them with a rebate for purchasing those items.

Although price is the key factor affecting consumer food choice, a majority of respondents (75%) also cited availability as the main reason for purchasing food at their chosen vendors or malls given the convenience, echoing results of other studies [53, 54]. Poorer eating habits among low-income populations is connected to obesogenic neighbourhood environments where access to healthy foods is limited and the concentration of convenience stores is high [55–57]. Identifying food patterns that are nutrient-rich, affordable, easily accessible, and appealing should be a priority for the government and community-based organisations in order to provide another avenue for altering the food environment of low-income communities in urban areas.

This study included a large sample of low-income residents in urban Kuala Lumpur with diversity in age and ethnicity who met face-to-face with trained interviewers. However, several limitations need to be taken into consideration when interpreting the study findings. Firstly, proxy response bias could not be ruled out as we used low-cost housing programmes as proxies to target the urban poor populations. Study findings may not be applicable across heterogenous urban community settings. All data was self-reported. We did not assess all possible factors affecting food choice, including advertising and cultural preferences. Validity may be affected by social desirability and recall bias. However, we minimized the bias by encouraging respondents to be forthright and explaining the dietary questions with common food examples.

## Conclusions

Adults in low-cost housing communities have unhealthy dietary patterns with low intake of fruits and vegetables and high intake of ultra-processed foods and beverages as well as high-calorie local foods, which increase risks of obesity and metabolic-related disorders. These findings highlight the urgent need for effective healthy food pricing and nutrition-related interventions which respond to the specific dietary practices and needs of Malaysia’s urban B40 population. Reducing the risk of obesity in Malaysia will require educating low-income families on diet-disease relationships and possibilities for inexpensive, healthy eating that rely on minimally processed fresh foods. Policymakers engaging the food industry are advised to consider how to increase the affordability and availability of healthy foods in low-income communities.

## Supplementary Information

**Additional file 1: Supplementary Table 1.** Gender and age groups of respondents by study locations.

## Data Availability

Data are available upon reasonable request. Data used for this study can be accessed upon request from the corresponding author.

## Abbreviations

B40: Households in the bottom 40% of the economic spectrum
MOH: Ministry of Health
NCDs: Noncommunicable diseases
SSBs: Sugar-sweetened beverages
WHO: World Health Organization

## References

[1] Organization WH (2016). World Health Organization obesity and overweight fact sheet.

[2] Ford ND, Patel SA, Narayan KMV (2017). Obesity in low- and middle-income countries: burden, drivers, and emerging challenges. Annu Rev Public Health.

[3] Gakidou E, Ng M, Fleming T, Robinson M, Thomson B, Graetz N (2014). Global, regional and national prevalence of overweight and obesity in children and adults 1980–2013: a systematic analysis. Lancet..

[4] Helble M, Francisco K (2017). The imminent obesity crisis in Asia and the Pacific: first cost estimates.

[5] Institute for Public Health (2020). National health and morbidity survey (NHMS) 2019: non-communicable diseases, healthcare demand and health literacy.

[6] Institute for Public Health (2012). National Health and morbidity survey (NHMS) 2011: non-communicable diseases.

[7] Popkin BM, Adair LS, Ng SW (2012). Global nutrition transition and the pandemic of obesity in developing countries. Nutr Rev.

[8] Hruby A, Hu FB (2015). The epidemiology of obesity: a big picture. Pharmacoeconomics..

[9] Kanter R, Caballero B (2012). Global gender disparities in obesity: a review. Adv Nutr.

[10] Andoy-Galvan JA, Lugova H, Patil SS, Wong YH, Baloch GM, Suleiman A, et al. Income and obesity in an urban poor community: a cross-sectional study. F1000research. 2020;9:160. 10.12688/f1000research.22236.1 PMC7194455 32399203

[11] Kataria I, Ngongo C, Lim SC, Kocher E, Kowal P, Chandran A (2020). Development and evaluation of a digital, community-based intervention to reduce noncommunicable disease risk in a low-resource urban setting in Malaysia: a research protocol. Implementation science communications.

[12] Malaysia DS (2019). Household income & basic amenities survey report 2019.

[13] Malaysia DoS. Household Income & Basic Amenities Survey Report 2019. Putrajaya. Malaysia: Department of Statistics; 2020.

[14] Department of Statistics Malaysia (2010). Population and housing census of Malaysia 2010.

[15] Bonita R, De Courten M, Dwyer T, Jamrozik K, Winkelmann R. Surveillance of risk factors for noncommunicable diseases: the WHO STEPwise approach: summary. Geneva: Noncommunicable Diseases and Mental Health, World Health Organization; 2001.

[16] Monteiro CA, Cannon G, Levy RB, Moubarac J-C, Louzada ML, Rauber F, Khandpur N, Cediel G, Neri D, Martinez-Steele E (2019). Ultra-processed foods: what they are and how to identify them. Public Health Nutr.

[17] Monteiro CA, Cannon G, Lawrence M, Costa Louzada MD, Pereira Machado P (2019). Ultra-processed foods, diet quality, and health using the NOVA classification system.

[18] Boeing H, Bechthold A, Bub A, Ellinger S, Haller D, Kroke A (2012). Critical review: vegetables and fruit in the prevention of chronic diseases. Eur J Nutr.

[19] Bazzano LA, He J, Ogden LG, Loria CM, Vupputuri S, Myers L (2002). Fruit and vegetable intake and risk of cardiovascular disease in US adults: the first National health and nutrition examination survey epidemiologic follow-up study. Am J Clin Nutr.

[20] Pomerleau J, Joint F, Organization WH. Effectiveness of interventions and programmes promoting fruit and vegetable intake [electronic resource]. Geneva: World Health Organization; 2005.

[21] Izzah AN, Aminah A, Pauzi AM, Lee Y, Rozita WW, Fatimah DS (2012). Patterns of fruits and vegetable consumption among adults of different ethnics in Selangor, Malaysia. Int Food Res J.

[22] Pei CS, Appannah G, Sulaiman N (2018). Household food insecurity, diet quality, and weight status among indigenous women (Mah Meri) in peninsular Malaysia. Nutr Res Pract.

[23] Lee SJ, Ryu HK (2018). Relationship between dietary intakes and the double burden of malnutrition in adults of Malang, Indonesia: an exploratory study. Nutr Res Pract.

[24] Niessen LW, Mohan D, Akuoku JK, Mirelman AJ, Ahmed S, Koehlmoos TP (2018). Tackling socioeconomic inequalities and non-communicable diseases in low-income and middle-income countries under the sustainable development agenda. Lancet.

[25] Blisard N, Stewart H, Jolliffe D (2004). Low-income households'expenditures on fruits and vegetables.

[26] Yen ST, Tan AK, Feisul MI (2015). Consumption of fruits and vegetables in Malaysia: profiling the daily and nondaily consumers. Asia Pac J Public Health.

[27] Irala-Estévez JD, Groth M, Johansson L, Oltersdorf U, Prättälä R, Martinez-Gonzalez MA (2000). A systematic review of socio-economic differences in food habits in Europe: consumption of fruit and vegetables. Eur J Clin Nutr.

[28] Daud NM, Fadzil NI, Yan LK, Makbul IAA, Yahya NFS, Teh AH, et al. Knowledge, attitude and practice regarding dietary fibre intake among Malaysian rural and urban adolescents. Malays J Nutr. 2018;24(1):77–88.

[29] Okuda N, Miura K, Okayama A, Okamura T, Abbott RD, Nishi N, Fujiyoshi A, Kita Y, Nakamura Y, Miyagawa N, Hayakawa T (2015). Fruit and vegetable intake and mortality from cardiovascular disease in Japan: a 24-year follow-up of the NIPPON DATA80 study. Eur J Clin Nutr.

[30] Kjøllesdal M, Htet AS, Stigum H, Hla NY, Hlaing HH, Khaine EK, Khaing W, Khant AK, Khin NO, Mauk KK, Moe EE (2016). Consumption of fruits and vegetables and associations with risk factors for non-communicable diseases in the Yangon region of Myanmar: a cross-sectional study. BMJ Open.

[31] Fogelholm M, Anderssen S, Gunnarsdottir I, Lahti-Koski M (2012). Dietary macronutrients and food consumption as determinants of long-term weight change in adult populations: a systematic literature review. Food Nutr Res.

[32] Shi Z, Hu X, Yuan B, Hu G, Pan X, Dai Y, Byles JE, Holmboe-Ottesen G (2008). Vegetable-rich food pattern is related to obesity in China. Int J Obes.

[33] Gibney MJ (2019). Ultra-processed foods: definitions and policy issues. Curr Dev Nutr.

[34] Rauber F, da Costa Louzada ML, Steele EM, Millett C, Monteiro CA, Levy RB (2018). Ultra-processed food consumption and chronic non-communicable diseases-related dietary nutrient profile in the UK (2008–2014). Nutrients..

[35] Julia C, Martinez L, Allès B, Touvier M, Hercberg S, Méjean C, Kesse-Guyot E (2018). Contribution of ultra-processed foods in the diet of adults from the French NutriNet-Santé study. Public Health Nutr.

[36] Juul F, Martinez-Steele E, Parekh N, Monteiro CA, Chang VW (2018). Ultra-processed food consumption and excess weight among US adults. Br J Nutr.

[37] Monteiro CA, Moubarac JC, Cannon G, Ng SW, Popkin B (2013). Ultra-processed products are becoming dominant in the global food system. Obes Rev.

[38] Monteiro CA, Cannon G (2012). The impact of transnational “big food” companies on the south: a view from Brazil. PLoS Med.

[39] Poti JM, Mendez MA, Ng SW, Popkin BM (2016). Highly processed and ready-to-eat packaged food and beverage purchases differ by race/ethnicity among US households. J Nutr.

[40] Cheah YK, Adzis AA, Bakar JA, Applanaidu SD (2019). Factors associated with consumption of sugar-sweetened foods and beverages in Malaysia: an ethnic comparison. Int J Diabetes Dev Countries.

[41] Gordon NP, Hsueh L (2021). Racial/ethnic, gender, and age group differences in cardiometabolic risks among adults in a northern California health plan: a cross-sectional study. BMC Public Health.

[42] Devasahayam TW. Consumed with modernity and ‘tradition’: Food, women, and ethnicity in changing urban Malaysia. New York, United States: Syracuse University; 2001.

[43] Organization WH (2000). Healthy nutrition: the role of women: report on a WHO meeting, Murmansk, Russian Federation 14–15 June 2000.

[44] Bowers D (2000). Cooking trends echo changing roles of women. Food Review/Nat Food Rev.

[45] Mortimer G. Toward a shopping typology of primary male grocery shoppers. Int J Retail Distrib Manag. 2012;40:790–810.

[46] Gram M (2015). Buying food for the family: negotiations in parent/child supermarket shopping: an observational study from Denmark and the United States. J Contemp Ethnogr.

[47] Wijayaratne SP, Reid M, Westberg K, Worsley A, Mavondo F. Food literacy, healthy eating barriers and household diet. Eur J Mark. 2018;52(2):2449–77.

[48] Crane MM, Tangney CC, French SA, Wang Y, Appelhans BM (2019). Gender comparison of the diet quality and sources of food purchases made by urban primary household food purchasers. J Nutr Educ Behav.

[49] Wijayaratne S, Westberg K, Reid M, Worsley A (2021). A qualitative study exploring the dietary gatekeeper's food literacy and barriers to healthy eating in the home environment. Health Promot J Austral.

[50] Waterlander WE, de Haas WE, van Amstel I, Schuit AJ, Twisk JW, Visser M (2010). Energy density, energy costs and income–how are they related?. Public Health Nutr.

[51] Powell LM, Chaloupka FJ (2009). Food prices and obesity: evidence and policy implications for taxes and subsidies. Milbank Q.

[52] Williams LK, Abbott G, Thornton LE, Worsley A, Ball K, Crawford D (2014). Improving perceptions of healthy food affordability: results from a pilot intervention. Int J Behav Nutr Phys Act.

[53] D'Angelo H, Suratkar S, Song HJ, Stauffer E, Gittelsohn J (2011). Access to food source and food source use are associated with healthy and unhealthy food-purchasing behaviours among low-income African-American adults in Baltimore City. Public Health Nutr.

[54] Haynes-Maslow L, McGuirt J, Trippichio G, Armstrong-Brown J, Ammerman AS, Leone LA (2020). Examining commonly used perceived and objective measures of fruit and vegetable access in low-income populations and their association with consumption. Transl Behav Med.

[55] Hilmers A, Hilmers DC, Dave J (2012). Neighborhood disparities in access to healthy foods and their effects on environmental justice. Am J Public Health.

[56] Larson NI, Story MT, Nelson MC (2009). Neighborhood environments: disparities in access to healthy foods in the US. Am J Prev Med.

[57] Hosler AS, Varadarajulu D, Ronsani AE, Fredrick BL, Fisher BD (2006). Low-fat milk and high-fiber bread availability in food stores in urban and rural communities. J Public Health Manage Pract.

