# Supplementary material for: Dietary practices, food purchasing, and perceptions about healthy food availability and affordability: a cross-sectional study of low-income Malaysian adults
Source: BMC Public Health. 2022 Jan 28;22:192. doi: 10.1186/s12889-022-12598-y (PMC8795714; doi:10.1186/s12889-022-12598-y)
Supplement: Supplementary file 1 — Additional file 1: Supplementary Table 1. Gender and age groups of respondents by study locations. [file 12889_2022_12598_MOESM1_ESM.docx]

**Supplementary Table 1** Gender and age groups of respondents by study locations

| Gender | Age group (years) | Study Locations | | | | | |
| --- | --- | --- | --- | --- | --- | --- | --- |
|  |  | **PA Seri Kota** | **PA Sri Negeri Sembilan** | **PPR Pekan Kepong Setia** | **PPR Wahyu** | **PPR Fasa 8/9 Bandar Baru Sentul** | **PA Seri Melaka** |
| Male | 18-29 | 88 | 90 | 86 | 92 | 87 | 90 |
|  | 30-39 | 55 | 55 | 57 | 58 | 59 | 56 |
|  | 40-49 | 47 | 45 | 45 | 47 | 44 | 44 |
|  | 50-59 | 30 | 31 | 31 | 36 | 30 | 31 |
|  | 60+ | 28 | 28 | 25 | 31 | 25 | 26 |
| Female | 18-29 | 86 | 86 | 86 | 100 | 88 | 89 |
|  | 30-39 | 55 | 57 | 58 | 57 | 55 | 55 |
|  | 40-49 | 44 | 44 | 44 | 48 | 45 | 44 |
|  | 50-59 | 31 | 31 | 32 | 31 | 32 | 30 |
|  | 60+ | 26 | 27 | 25 | 29 | 27 | 24 |
| Total | | **490** | **494** | **489** | **529** | **492** | **489** |

*PA/PPR, People's Housing Programme*
